# Supplementary material for: Increased longevity due to sexual activity in mole-rats is associated with transcriptional changes in the HPA stress axis
Source: eLife. 2021 Mar 16;10:e57843. doi: 10.7554/eLife.57843 (PMC8012063; doi:10.7554/eLife.57843)
Supplement: Source data 2. — (a) Enrichment analysis results for status-dependent gene expression on Kyoto Encyclopedia of Genes and Genomes pathways. The data set contains one *.tsv file per analyzed tissue, as well as an additional *.tsv file for the weighted cross-tissue level results. (b) (zip) Enrichment analysis results for status-dependent gene expression on Molecular Signatures Database hallmarks. The data set contains one *.tsv file per analyzed tissue, as well as an additional *.tsv file for the weighted cross-tissue level results. (c) (tsv.gz) Overview of the weighted cross-tissue differential gene expression analysis. Contains all p-values, fold-changes, and mean expression values for all genes across all tissues as well as the weighted, combined cross-tissue test statistics, p-values, and fold-changes for all genes. (d) (tsv) Genes of the Digital Aging Atlas used in this study. (e) (zip) Comparison of status-dependent differentially expressed genes with results of an earlier, similar study involving naked mole-rats and guinea pigs. The data set contains one *.tsv file for each of the 10 tissues that were examined in both studies. Each *.tsv file lists the differentially expressed genes for the respective tissue as identified in this study with the determined false discovery rates and fold-changes, as well as the fold-changes determined in the earlier study using naked mole-rats and guinea pigs. (f) (zip) Glucocorticoid receptor target genes that were determined by Phuc Le et al. and tested in this study for enrichment of status-dependent differential gene expression. The data set contains one *.tsv file each for target genes determined via chromatin immunoprecipitation and differential gene expression analysis. Phuc Le et al. determined differentially expressed genes between mice that were treated with exogenous glucocorticoids and untreated controls. The relevant table columns from Phuc Le et al. were added by human gene symbol and Entrez IDs that were used for enrichment analysis [file elife-57843-data2.zip › Data_2h.zip!/Skin/mechowii_breeder_VS_non-breeder.html]

BatchQC Report


# BatchQC Report

Tests for checking Batch Effects

- Contents
- BatchQC Report

- Summary
  - Confounding
  - Variation Analysis
  - P-value Analysis
- Differential Expression
  - Expression Plot
  - LIMMA
- Median Correlations
- Heatmaps
  - Heatmap
  - Sample Correlations
- Circular Dendrogram
- PCA: Principal Component Analysis
  - PCA
  - Explained Variation
- Shape
- Combat Plots
- SVA
  - Summary

# BatchQC Report

### 2021-01-23

# Summary

## Confounding

### Number of samples in each Batch and Condition

|  | Batch 151110 | Batch 170208 | Batch 170217 |
| --- | --- | --- | --- |
| **Condition crowned** | 12 | 0 | 0 |
| **Condition worker** | 6 | 2 | 2 |

### Measures of confounding between Batch and Condition

|  | Standardized Pearson Correlation Coefficient | Cramer’s V |
| --- | --- | --- |
| **Confounding Coefficients (0=no confounding, 1=complete confounding)** | 0.6489 | 0.5164 |

## Variation Analysis

### Variation explained by Batch and Condition

|  | Full (Condition+Batch) | Condition | Batch |
| --- | --- | --- | --- |
| **Min.** | 0 | 0 | 0 |
| **1st Qu.** | 5.152 | 0.318 | 2.843 |
| **Median** | 10.67 | 1.385 | 6.483 |
| **Mean** | 13.74 | 2.893 | 9.768 |
| **3rd Qu.** | 19.33 | 3.915 | 13.32 |
| **Max.** | 98.86 | 47.04 | 98.85 |

## P-value Analysis

### Distribution of Batch and Condition Effect p-values Across Genes

|  | Min. | 1st Qu. | Median | Mean | 3rd Qu. | Max. | Ps<0.05 |
| --- | --- | --- | --- | --- | --- | --- | --- |
| **Batch P-values** | 0 | 0.2095 | 0.4751 | 0.4765 | 0.7363 | 1 | 0.07865 |
| **Condition P-values** | 7.369e-05 | 0.3031 | 0.5689 | 0.5443 | 0.7947 | 1 | 0.03453 |

# Differential Expression

## Expression Plot

Boxplots for all values for each of the samples and are colored by batch membership.

## LIMMA

|  | Condition: worker (logFC) | AveExpr | t | P.Value | adj.P.Val | B |
| --- | --- | --- | --- | --- | --- | --- |
| **PLXNA4** | 178.3 | 181.2 | 5.177 | 5.704e-05 | 0.3429 | -4.538 |
| **ALDH3B1** | 173.8 | 427.1 | 5.076 | 7.122e-05 | 0.3429 | -4.539 |
| **SMIM10** | 121.4 | 179.5 | 5.074 | 7.148e-05 | 0.3429 | -4.539 |
| **ADAMTS14** | 86.42 | 124.1 | 4.84 | 0.00012 | 0.4319 | -4.541 |
| **HDAC5** | 219.1 | 642 | 4.485 | 0.0002653 | 0.5775 | -4.545 |
| **KIF5C** | 54.92 | 63.68 | 4.46 | 0.0002805 | 0.5775 | -4.546 |
| **ITPR2** | 1548 | 3551 | 4.459 | 0.0002809 | 0.5775 | -4.546 |
| **DDX59** | 200.7 | 950.6 | 4.331 | 0.0003749 | 0.6603 | -4.547 |
| **ZCCHC12** | 286.5 | 290.7 | 4.288 | 0.0004129 | 0.6603 | -4.548 |
| **TNFRSF18** | 192.7 | 311.4 | 4.156 | 0.0005571 | 0.7365 | -4.549 |

# Median Correlations

This plot helps identify outlying samples.

# Heatmaps

## Heatmap

This is a heatmap of the given data matrix showing the batch effects and variations with different conditions.

## Sample Correlations

This is a heatmap of the correlation between samples.

# Circular Dendrogram

This is a Circular Dendrogram of the given data matrix colored by batch to show the batch effects.

# PCA: Principal Component Analysis

## PCA

This is a plot of the top two principal components colored by batch to show the batch effects.

## Explained Variation

|  | Proportion of Variance (%) | Cumulative Proportion of Variance (%) | Percent Variation Explained by Either Condition or Batch | Percent Variation Explained by Condition | Condition Significance (p-value) | Percent Variation Explained by Batch | Batch Significance (p-value) |
| --- | --- | --- | --- | --- | --- | --- | --- |
| **PC1** | 28.6 | 28.6 | 6.6 | 1.9 | 0.9029 | 6.5 | 0.6412 |
| **PC2** | 19.04 | 47.64 | 8 | 0.5 | 0.8277 | 7.8 | 0.4908 |
| **PC3** | 13.05 | 60.68 | 32.6 | 4.3 | 0.04903 | 15.9 | 0.04252 |
| **PC4** | 6.972 | 67.66 | 14.2 | 0.4 | 0.6041 | 12.9 | 0.2604 |
| **PC5** | 5.97 | 73.62 | 19.2 | 0 | 0.4756 | 16.8 | 0.1474 |
| **PC6** | 4.073 | 77.7 | 5.1 | 3 | 0.4284 | 1.7 | 0.8167 |
| **PC7** | 2.898 | 80.6 | 31.1 | 0 | 0.2343 | 25.3 | 0.0352 |
| **PC8** | 2.355 | 82.95 | 29.5 | 26.3 | 0.02701 | 6.8 | 0.6713 |
| **PC9** | 1.921 | 84.87 | 24.4 | 18.9 | 0.2316 | 17.9 | 0.5355 |
| **PC10** | 1.712 | 86.58 | 20 | 8.2 | 0.2665 | 14.2 | 0.2892 |
| **PC11** | 1.663 | 88.24 | 0.7 | 0.3 | 0.91 | 0.6 | 0.9595 |
| **PC12** | 1.482 | 89.73 | 7.8 | 2.5 | 0.3142 | 2.3 | 0.6065 |
| **PC13** | 1.386 | 91.11 | 11.8 | 10.3 | 0.3462 | 7.2 | 0.8605 |
| **PC14** | 1.34 | 92.45 | 23.1 | 1.9 | 0.2761 | 17.7 | 0.1119 |
| **PC15** | 1.261 | 93.71 | 1.1 | 0 | 0.8849 | 0.9 | 0.9093 |
| **PC16** | 1.19 | 94.9 | 9.8 | 2.9 | 0.5892 | 8.3 | 0.5151 |
| **PC17** | 1.131 | 96.04 | 9.6 | 8.7 | 0.2428 | 2.3 | 0.9161 |
| **PC18** | 1.105 | 97.14 | 33.9 | 2.6 | 0.845 | 33.7 | 0.03051 |
| **PC19** | 1.063 | 98.2 | 4.8 | 3.9 | 0.3604 | 0.1 | 0.9228 |
| **PC20** | 0.9279 | 99.13 | 4.7 | 3 | 0.3863 | 0.5 | 0.8535 |
| **PC21** | 0.8687 | 100 | 2.1 | 0.5 | 0.6013 | 0.6 | 0.8639 |
| **PC22** | 2.461e-29 | 100 | 15.3 | 5.9 | 0.812 | 15 | 0.3892 |

# Shape

This is a heatmap plot showing the variation of gene expression mean, variance, skewness and kurtosis between samples grouped by batch to see the batch effects variation

```
## Note: Sample-wise p-value is calculated for the variation across samples on the measure across genes. Gene-wise p-value is calculated for the variation of each gene between batches on the measure across each batch. If the data is quantum normalized, then the Sample-wise measure across genes is same for all samples and Gene-wise p-value is a good measure.
```

# Combat Plots

This is a plot showing whether parametric or non-parameteric prior is appropriate for this data. It also shows the Kolmogorov-Smirnov test comparing the parametric and non-parameteric prior distribution.

```
## Found 3 batches
## Adjusting for 1 covariate(s) or covariate level(s)
## Standardizing Data across genes
## Fitting L/S model and finding priors
```

```
## Warning in ks.test(gamma.hat[1, ], "pnorm", gamma.bar[1], sqrt(t2[1])): ties should not be present for the Kolmogorov-Smirnov test
```

```
## Warning in ks.test(gamma.hat[1, ], "pnorm", gamma.bar[1], sqrt(shinyInput$t2[1])): ties should not be present for the Kolmogorov-Smirnov test
```

```
## Warning in ks.test(delta.hat[1, ], invgam): p-value will be approximate in the presence of ties
```

```
## Batch mean distribution across genes: Normal vs Empirical distribution
## Two-sided Kolmogorov-Smirnov test
## Selected Batch: 1
## Statistic D = 0.01529
## p-value = 0.0024
## 
## 
## Batch Variance distribution across genes: Inverse Gamma vs Empirical distribution
## Two-sided Kolmogorov-Smirnov test
## Selected Batch: 1
## Statistic D = 0.1781
## p-value = 0Note: The non-parametric version of ComBat takes much longer time to run and we recommend it only when the shape of the non-parametric curve widely differs such as a bimodal or highly skewed distribution. Otherwise, the difference in batch adjustment is very negligible and parametric version is recommended even if p-value of KS test above is significant.
```

# SVA

## Summary

```
## Number of Surrogate Variables found in the given data: 0
```

Questions? Kindly contact `Solaiappan Manimaran` `mani2012 [at] bu.edu` using the subject heading `BatchQC question`.
